# Investigation on Periodically Surface-Corrugated Long-Period Gratings Inscribed on Photonic Crystal Fibers

**DOI:** 10.1186/s11671-017-1968-1

**Published:** 2017-04-04

**Authors:** Young-Geun Han

**Affiliations:** Department of Physics and the Research Institute for Research Institute for Natural Sciences, Hanyang University, 222 Wangsimni-ro, Seongdong-gu, Seoul 133-791 South Korea

**Keywords:** Long-period gratings, Photonic crystal fiber, Strain sensor, Temperature-insensitivity

## Abstract

Transmission characteristics of periodically surface-corrugated long-period gratings (LPGs) inscribed on photonic crystal fibers (PCFs) using a wet-etching technique were experimentally investigated. A conventional wet method was implemented to periodically engrave the silica cladding region of the PCFs resulting in the periodic surface corrugation in the PCF. After applying the external strain to the PCF with the periodic surface micro-ridges, periodic modulation of refractive index based on the photoelastic effect is induced resulting in the formation of the PCF-based LPG. Increasing the applied strain successfully improves the extinction ratio of the resonant peak of the PCF-based LPG without the resonant wavelength shift. We also measured the transmission characteristics of the PCF-based LPG with variations in temperature and ambient index.

## Background

Long-period fiber gratings (LPGs) have been of interest to optical communication systems and optical sensors because of their various advantages, such as wavelength-selective nature, mass production, compatibility, easy installation, and electromagnetic immunity [1]. The LPGs based on the periodic modulation of the refractive index in the core region of the conventional single-mode fiber (SMF) can couple the fundamental core mode to cladding modes [1, 2]. Since the cladding modes is readily affected by external perturbation change, like strain, temperature, bending, and ambient index, the LPG has usually been exploited to realize highly sensitive fiber-optic sensors. With a conventional SMF with the germanium-doped core, essentially, periodic exposures of the SMF to UV laser is capable of inducing refractive index change in the core resulting in the formation of LPGs [1]. However, the photo-induced refractive index change is not usually applicable to fabricate the LPG if optical fibers like silica fibers or photonic crystal fibers (PCFs) have no photosensitivity. The PCF typically has the periodic structure of axially aligned air holes in the silica cladding along the entire fiber length [3]. The PCF has many advantages, such as the endless flexibility in design and fabrication, low nonlinearities, and so on [3]. Since the PCF is typically composed of pure silica and air holes along the fiber length, it is impossible to induce the UV-induced photo-refractive index change. To fabricate LPGs using PCFs, structural deformation technique with CO_2_ laser has been exploited [4–6]. The femtosecond laser-induced LPG after filling cladding holes in the PCF was also reported [7]. One of the drawbacks in the previous methods, however, was asymmetrical or one-side deformation of the surface of the PCF. To fabricate LPGs with symmetric deformation, a mirror-assisted symmetric exposure technique with CO_2_ laser was proposed [8]. In this paper, we investigate on the transmission characteristics of the LPG based on the PCF. The azimuthally symmetric and periodic micro-ridges on the surface of the silica cladding in the PCF are successfully produced by using a wet-etching technique. Transmission characteristics of the periodically surface-corrugated LPGs inscribed on the PCF are observed. The applied strain effectively changes the transmission characteristics of the proposed PCF-based LPGs because of photoelastic effect. Increasing temperature makes the extinction ratio of the proposed PCF-based LPG diminished because of the reduction of photoelastic effect. We also measure the transmission characteristics of the proposed PCF-based LPG with variations in ambient index.

## Experiments and Discussion

**Fig. 1:**
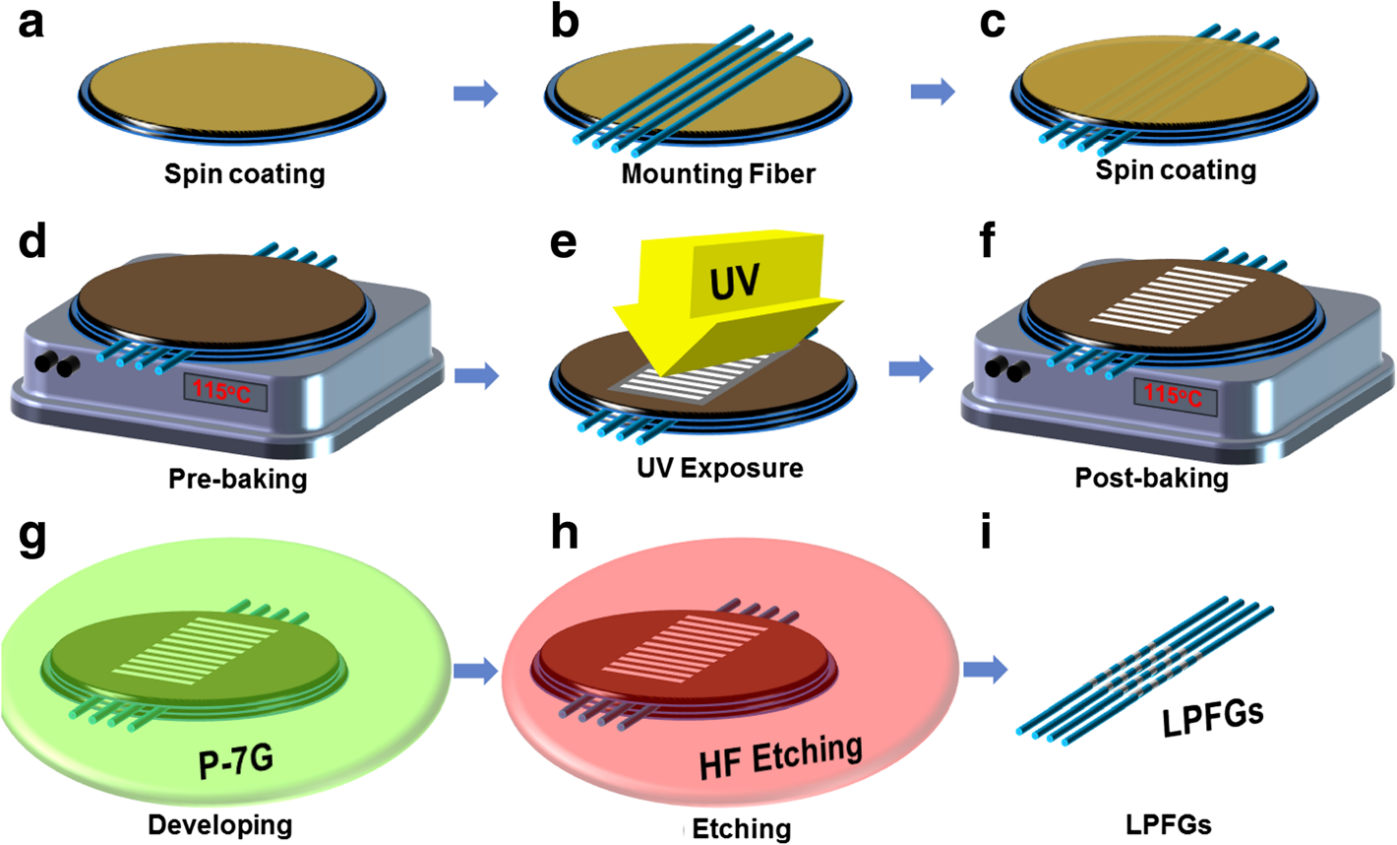
Fabrication procedure of the PCF-based LPG by using a wet etching technique. **a** spin coating, **b** mounting fiber, **c** spin coating, **d** pre-baking, **e** UV exposure, **f** post baking, **g** developing, **h** etching, and **i** fabrication of LPGs

Figure 1 shows the fabrication process of the PCF-based LPG by using a wet-etching method [9, 10]. In the proposed technique, we exploited the UV polymer to induce the azimuthally symmetric and periodic micro-ridges on the surface of the PCF. The UV polymer with a thickness of 120 μm was coated on the silicon substrate by using a spin coater. We put the PCF on the silicon substrate with the UV polymer and covered it by using the same UV polymer with the same thickness. During the pre-baking process in the hot plate at a temperature of 115 °C, the undesirable solvent in the UV polymer was vaporized. We prepared the sample based on the PCF that was entirely surrounded by the UV polymer as seen in Fig. 1. Then, we periodically exposed the sample to the UV lamp by using an amplitude mask with a grating period of 600 mm and a total length of 2 cm. The post-baking procedure was additionally required to completely eliminate the remaining solvent in the UV polymer in the sample. The polymer regions irradiated by the UV lamp in the sample were removed by using a developer resulting to the periodic and symmetric pattern of the polymer on the surface of the PCF. Then, the sample was soaked in hydrofluoric acid (HF) solution. The UV-polymer-patterned silica cladding regions of the PCF protected penetration of HF solution. Contrarily, the silica cladding sections of the PCF without the polymer coating were gradually engraved by HF solution. We disposed of the remained periodic polymer by using the acetate solution. Consequently, we successfully realized the PCF-based LPG with azimuthally symmetric and periodic micro-ridges on the surface of the PCF as seen in Fig. 1.

**Fig. 2:**
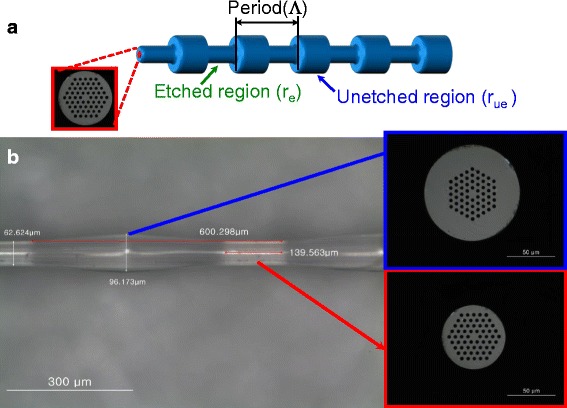
**a** Structure of the proposed PCF-based LPG. **b** Photograph of the fabricated PCF-based LPG measured by using an optical microscope

Figure 2a shows the schematic structure of the proposed PCF-based LPG. Figure 2b exhibits the photography of the surface of the fabricated LPG inscribed on the PCF measured by using an optical microscope. The azimuthally symmetric and periodic deformation in the PCF was evidently observed. The diameter of the PCF with and without corrosion was reduced and measured to be ~62.6 and 96.2 μm, respectively. The grating period was measured to be ~600 μm, which was consistent with that of the amplitude mask. The hexagonal array structure of air holes in the PCF should be maintained incipiently in the etched and the unetched regions as seen in Fig. 2b.

By considering periodic micro-ridges in the PCF, the cross-sections between the etched and the unetched cladding regions were apparently different because of the different amount of the remained silica cladding areas in the PCF. It means that the applied strain is capable of inducing different effective index change based on the photoelastic effect in the etched and the unetched regions with different diameters [9, 10]. Since the PCF in the experiment has the azimuthally symmetric and periodic micro-ridges, the periodic index modulation based on the photoelastic effect along the PCF length must be created by strain, which results in the formation of the PCF-based LPG. Consequently, mode coupling between the core and the cladding modes in the proposed PCF-based LPG successfully produces the harmonic resonant peaks in the transmission spectrum. The transmission (*T*) of the PCF-based LPG is critically changed by the applied strain, which can be theoretically described as [9, 10]

1 $$ T\cong { \cos}^2\left(\overline{\kappa}{l}_{\mathrm{pcf}}\right)={ \cos}^2\left[{\sigma}_e\left(\frac{{r_{ue}}^2}{{r_e}^2}-1\right)\varepsilon {l}_{\mathrm{pcf}}\right], $$

where $\overline{\kappa}$ is the average coupling coefficient. *l*_PCF_ is the total length of the PCF-based LPG. $\sigma$_*e*_ is the photoelastic coefficient. *r*_e_ and *r*_ue_ are the radii of the etched and the unetched cladding regions in the PCF, respectively. $\varepsilon$ is the applied strain. The proposed PCF-based LPG with the periodic structure of the surface corrugations has the particular index change based on photoelastic effect and intrinsically structural index change [9, 10]. Since the micro-ridges are periodically patterned in the silica of the PCF, the averaged effective refractive index of the cladding mode will play important role in the mode coupling between the core and the cladding modes and the variation of the periodic structural index of the core mode can be negligible [9, 10]. The resonant wavelength (*λ*_p_) of the proposed PCF-based LPG with periodic micro-ridges can be written as [9, 10]

2 $$ {\lambda}_p=\frac{\varLambda \left({n}_{co}-{\overline{n}}_{cl}\right)}{1+\left({\overline{\kappa}}_{cl}-{\kappa}_{co}\right)\frac{\varLambda}{2\pi}}, $$

where *Λ* is the grating period. *n*_*co*_ is the effective refractive index of the core mode. ${\overline{n}}_{cl}$ is the averaged effective index of the cladding mode. ${\overline{\kappa}}_{cl}$ and ${\kappa}_{{}_{co}}$ are the self-coupling coefficients of the average cladding mode and the core mode, respectively.

**Fig. 3:**
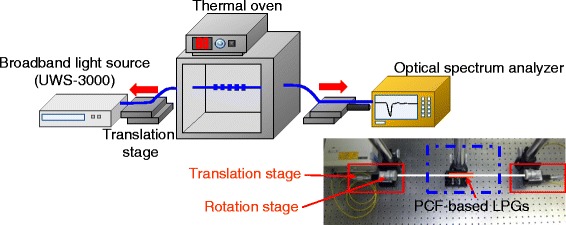
Experimental setup for measurement of the transmission characteristics of the PCF-based LPG with periodic surface corrugations

Figure 3 shows the experimental scheme for measurement of the transmission characteristics of the PCF-based LPG with periodic surface corrugations. The photograph for the experimental setup was shown in the inset. The measurement setup is composed of a broadband light source, linear translation and rotation stages, and an optical spectrum analyzer. Both ends of the PCF-LPG were positioned at two rotation stages on two linear translation stages. A distance between two stages was 30 cm. Strain was applied to the PCF-based LPG by moving the two linear translation stage outwards.

**Fig. 4:**
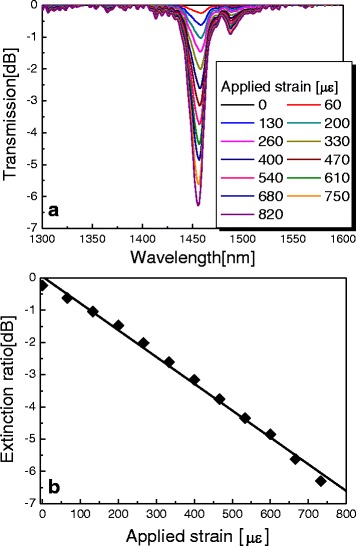
**a** Transmission spectra of the proposed PCF-based LPG with variations in strain. **b** Variation of extinction ratio as a function of strain

Figure 4a depicts the transmission spectra of the proposed PCF-based LPG with periodic micro-corrugations as the applied strain changes. When we applied strain to the proposed PCF-based LPG, the resonant peak resulting from the mode coupling between core and cladding modes based on the photoelastic effect was induced in the transmission spectrum. Increasing the applied strain makes the extinction ratio strong because of the improvement of the mode coupling based on the photoelastic effect [9, 10]. In Fig. 4b, the variation of extinction ratio was measured to be -6.89 when the applied strain was 800 με. However, the resonant wavelength was not severely shifted by the applied stain. Since the variations of the effective refractive indices based on the photoelastic effect in the core and the cladding regions are approximately the same, two self-coupling strengths in the core and the cladding modes are also equal [9, 10]. Therefore, the resonant wavelength of the PCF-based LPG was not critically changed by the applied strain.

**Fig. 5:**
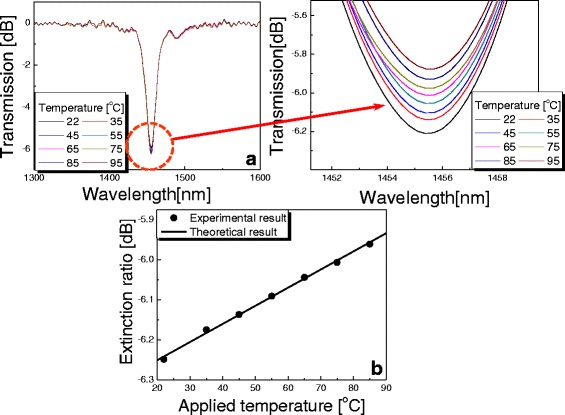
**a** Transmission spectra with variations in temperature. **b** Variation of extinction ratio as a function of temperature

Figure 5a exhibits the transmission spectra of the proposed PCF-based LPG with variations in temperature. The conventional LPG inscribed on the SMF has strong temperature dependence and the resonant wavelength must be shifted depending on the doping materials like germanium and boron in the core region [11, 12]. The resonant wavelength of the proposed PCF-based LPG with periodic micro-ridges, however, was not changed by temperature because the PCF was composed of a single material like silica only. As seen in Fig. 5b, the extinction ratio was reduced by temperature because the photoelastic effect was diminished by increasing temperature. Figure 5c shows theoretical and experimental results on the variation of extinction ratio of the proposed PCF-based LPG as a function of temperature. By considering Eq. (2) and thermos-optic coefficient of silica (0.55 × 10^−6^) [13], the variation of extinction ratio of the PCF-based LPG was theoretically analyzed. Extinction ratio of the PCF-based LPG was gradually decreased by temperature, which was measured to be −4.5 × 10^−3^ dB/°C. As seen in Fig. 5c, the theoretical result is in good agreement with the experimental one.

**Fig. 6:**
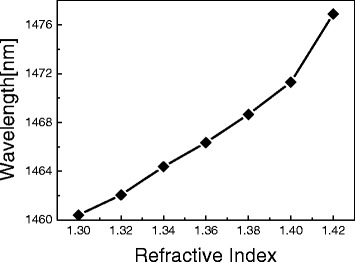
Resonant wavelength shift as a function of ambient index

Figure 6 shows the resonant wavelength shift of the PCF-based LPG as a function of ambient index. Increasing ambient index makes the resonant wavelength shift to longer wavelengths. Since the dispersion of the core mode in the PCF is higher than that of the cladding mode, ambient index affects the variation of the effective refractive index of the core mode more than that of the cladding mode [14]. Therefore, the resonant wavelength of the PCF-based LPG with periodic micro-ridges is shifted to longer wavelengths with variations in ambient index as seen in Fig. 6. The ambient index sensitivity of the proposed PCF-based LPG with periodic micro-ridges was measured to be ~108.9 nm/RIU.

## Conclusion

We fabricated the LPG based on the PCF by periodically etching the silica cladding in the PCF with a wet-etching technique. The azimuthally symmetric and periodic micro-corrugations were successfully patterned on the surface of the silica cladding of the PCF. The applied strain effectively induces the mode coupling between the core and the cladding modes based on the photoelastic effect, which results in the resonant peak in the transmission spectrum. Increasing strain makes the transmission peak depth of the PCF-based LPG with periodic micro-ridges strong because of the photoelastic effect. Since the photoelastic effect is reduced by temperature, the extinction ratio of the proposed PCF-based LPG was reduced by increasing temperature. The temperature sensitivity of the transmission was measured to be −4.5 × 10^−3^ dB/°C. We also measured the transmission characteristics of the proposed PCF-based LPG with variations in ambient index. In the PCF, the dispersion of the core mode is higher than that of the cladding mode. Since ambient index affects the variation of the effective refractive index of the core mode more than that of the cladding mode, increasing ambient index makes the resonant wavelength of the proposed PCF-based LPG shift to longer wavelengths. The ambient index sensitivity was measured to be ~108.9 nm/RIU. We believe that the experimental results are very useful for many applications to optical communications, fiber-optic sensors, instrument measurement, etc.
